# Adverse Collateral Effects of COVID-19 Public Health Restrictions on Physical Fitness and Cognitive Performance in Primary School Children

**DOI:** 10.3390/ijerph182111099

**Published:** 2021-10-22

**Authors:** Camille Chambonnière, Nicole Fearnbach, Léna Pelissier, Pauline Genin, Alicia Fillon, Audrey Boscaro, Line Bonjean, Mélina Bailly, Julie Siroux, Terry Guirado, Bruno Pereira, David Thivel, Martine Duclos

**Affiliations:** 1 Laboratory of Metabolic Adaptations to Exercise under Physiological and Pathological Conditions, Clermont Auvergne University, 63000 Clermont-Ferrand, France; camille.chambonniere@uca.fr (C.C.); fillonalicia@gmail.com (A.F.); audrey.boscaro@uca.fr (A.B.); line.bonjean@etu.uca.fr (L.B.); melina.bailly@uca.fr (M.B.); julie.siroux@etu.uca.fr (J.S.); terry.guirado@uca.fr (T.G.); david.thivel@uca.fr (D.T.); 2 Clinical Sciences Division, Pennington Biomedical Research Center, Baton Rouge, LA 70808, USA; nicole.fearnbach@pbrc.edu; 3 National Observatory for Physical Activity and Sedentary Behaviors, 63000 Clermont-Ferrand, France; p.genin@onaps.fr (P.G.); mduclos@chu-clermontferrand.fr (M.D.); 4 Biostatistics Unit, Clermont-Ferrand University Hospital, 63000 Clermont-Ferrand, France; bpereira@chu-clermontferrand.fr; 5 Clermont Auvergne University Foundation, 63000 Clermont-Ferrand, France; 6 UFR Medicine, Clermont Auvergne University, 63000 Clermont-Ferrand, France; 7 INRA, UMR 1019, 63000 Clermont-Ferrand, France; 8 Department of Sport Medicine and Functional Explorations, Clermont-Ferrand University Hospital, G. Montpied Hospital, 63000 Clermont-Ferrand, France

**Keywords:** COVID-19, physical fitness, executive function, confinement, children

## Abstract

The aim of the present study was to evaluate whether the COVID-19-related confinement and social restrictions affected the levels of physical fitness and academic achievement in primary school French children. A total of 206 primary school children (106 before confinements and 100 after restrictions) completed a test battery evaluating their anthropometric characteristics, body compositions, activity preferences, cognitive performances and physical fitness. The performance of the Standing Long Jump was better at T0 (169.9 ± 142.5 cm) compared to T1 (135.2 ± 31.4 cm) (*p* = 0.0367), and the Medicine Ball Throw performance declined from T0 to T1 (297.3 ± 81.1 cm vs. 249 ± 52 cm; *p* < 0.0001). Motor skills (26.9 ± 6.2 s vs. 30.9 ± 5.4 s; *p* < 0.0001), the shuttle-run test (stages completed), Maximal Aerobic Speed, and the estimated VO_2max_ were lower at T1 compared to T0 (*p* < 0.0001). Executive functioning was found to be greater at T0 compared to T1 (*p* < 0.0001). Explicit liking or wanting for sedentary or physical activities did not change between T0 and T1. Both overall physical fitness and cognitive performance drastically declined among primary school French children with the COVID-19-related public health restrictions, which reinforces the need to urgently develop preventive strategies in anticipation of further mitigation measures.

## 1. Introduction

After its appearance in Wuhan, China, in December 2019, the worldwide transmission of the SARS-CoV-2 virus (COVID-19) led to an unprecedented public health crisis, leading the World Health Organization (WHO) officially declaring a global pandemic on 11 March 2020. In the span of about 7 days, the progression of the virus led to global school closures in order to reduce the amount of social contact between students. Nearly 850 million children and adolescents were subjected to stay-at-home orders and engaged in virtual learning [1,2,3]. In France, a general national confinement was imposed on 17 March 2020, for a total of 55 days, with all primary and secondary schools closed and all teaching activities conducted online. Although the restrictions were removed in May 2020, protective measures and preventive precautions, which encouraged social distancing, were maintained throughout 2020.

As a direct consequence, the movement behaviors of both adults and youth have been affected and a plethora of international publications have clearly described an alarming decline in physical activity levels, with a simultaneous increase in sedentary behaviors (SED) in children and adolescents [4]. While these COVID-19-related alterations of PAL and SED time in youth have been associated with impaired mental, physical, and metabolic health and overall well-being [5,6,7,8,9], few results are available regarding the impact on objectively measured physical fitness. In a recent work, Wahl-Alexander and Camic assessed 9- to 14-year-old children’s cardiorespiratory fitness (20 m Progressive Aerobic Cardiovascular Endurance Run PACER) and muscle strength (curl-up and push-up tests) between August 2019 and July 2020 [10]. Although their baseline evaluation occurred about 7 months before the lockdown itself, the authors observed a significant decline in both cardiovascular endurance (26.7%) and muscle strength (20% decline at the push-up test and 35.6% decline at the sit-up test) in both boys and girls [10]. Lopez-Bueno et al. [11] also assessed cardiovascular fitness among 89 adolescent boys and girls aged 13.3 ± 0.9 years old using the multi-stage 20 m shuttle-run test, which showed that only girls experienced a significant decrease in estimated VO_2max_. However, the data for this study were collected between November 2019 and November 2020, which does not exclusively isolate the lockdown period. More recently, Jarnig and colleagues reported a decreased cardiorespiratory fitness among 764 7- to 10-year-old Austria children, all of whom exhibited a significantly increased body mass index [12]. These few studies provide, to our knowledge, the only available evidence regarding the potential effect of the COVID-19 period on objectively assessed fitness in youth. Given that fitness is associated with children’s leisure time activity preferences [13], it is important to investigate whether declines in fitness during the COVID-19 pandemic are associated with a greater desire to be sedentary, as this may pose a barrier to intervention.

Little is known regarding the effect of the COVID-19 confinement on academic achievement and executive functioning in children, while the imposed public health precautions kept the kids away from face-to-face teaching. In young adults, the lockdown negatively impacted executive function, particularly among individuals with pre-existing deficits [14]. In children, the early data from online-learning platforms indicate a drop in completed coursework [15] and an increased dispersion of test scores [16]. In their recent landmark study based on a database involving about 350,000 children aged 8- to 11-year- old from the Netherlands, Engzell and collaborators clearly underlined that children made little or no progress while learning from home during the confinement compared to a normal academic year [17]. Importantly, this learning loss was more pronounced among kids from disadvantaged homes [17]. Such cognitive and academic declines can be attributed to the stress induced by the lockdown conditions and the pandemic situation, the lower intellectual stimulation during virtual classes, and the increased sedentary time, especially screen time [18,19,20]. We hypothesize that a decline in physical fitness could also be involved, based on previous studies of the association between fitness and academic achievement in children and adolescents [21,22].

In this context, the aim of the present study is to evaluate whether the COVID-19-related confinement and social restrictions affected the levels of physical fitness and academic achievement in primary school French children. Using a forced-choice paradigm to understand implicit activity preferences [13], we also questioned the effect of this specific lockdown period on primary school children’s desires to be sedentary vs. physically active.

## 2. Materials and Methods

### 2.1. Population and Design

This study is part of the French National Observatory for Physical Activity and Sedentary Behaviors (ONAPS) national survey evaluating the impact of the 2020 COVID-19-related confinement. The baseline evaluations were taken in February 2020 (T0), right before the lockdown and the intervention that was supposed to last from March to May 2020 was cancelled. The same test battery was then conducted among children in 3rd and 4th grade after the lockdown and end of restrictions limiting social interactions in France (January 2021, T1).

A total of 206 primary school children from 3rd and 4th grades participated, 106 at T0 and 100 in T1. Children completed a test battery evaluating their anthropometric characteristics, body compositions, activity preferences, cognitive performances and physical fitness. Both parents and children received initial and adapted information sheets and signed consent forms as required by local ethical regulations (CPP Sud est VI. reference 2020/CE 27).

### 2.2. Anthropometric Characteristics and Body Composition

Body mass weight (kg) and height (cm) were measured by using a standard apparatus (Seca, Lea Mureaux), to the nearest 0.1 kg and 0.5 cm, respectively. Body mass index (BMI) was calculated as body weight divided by height squared (kilogram per square meter). Children were classified as having obesity (OG, [95th–99th] percentile) or extreme obesity (EOG, >99th percentile), according to BMI curves, chronological age and sex (Centers for Disease Control and Prevention—CDC) [23]. Body composition was assessed by bioelectrical impedance using a Tanita MC-780 multi-frequency, segmental body-composition analyzer. This consisted of a stand-alone unit which the participant had to step on with bare feet (standard mode). Information about the participant (age, sex, and height) was entered by the experimenter. Once body mass had been assessed by the Tanita scale, the participant had to take grips in both hands (alongside his/her body) during the impedance measurement (hand to foot). A full segmental analysis was performed in less than 20 s. This technology has been validated in children of various adiposity levels, showing a good level of validity and sensitivity to changes [24,25].

### 2.3. Executive Function

Executive function was assessed through the Trail Making Test (TMT), a 5 min pen-and-paper test with two sub-parts (A and B). The TMT-A evaluates the speed of treatment and attention while the TMT-B evaluates mental flexibility and executive functioning [26]. The TMT-A required children to connect, as quickly as possible, circles numbered from 1 to 25 in ascending order. The TMT-B contains circles, numbers and letters (from 1 to 25 and from A to Y) and the children were asked to connect them as quickly as possible in ascending order (e.g., 1-A, 2-B, 3-C). The time taken to complete each task (in seconds) and the number of mistakes was noted. This test has been validated in children [27].

### 2.4. Physical Fitness

#### 2.4.1. Cardiorespiratory Fitness

The 20 m shuttle-run test developed by Léger et al. was used as an indirect measurement of aerobic fitness [28]. This test is based on the linear relationship between the increase in oxygen consumption and the increase in running speed. The reliability of this test in both children and adults for the determination of maximum oxygen uptake has already been described [29]. During the test, participants were instructed to run as long as possible between two lines drawn 20 m apart at an increasing speed, which was imposed by a recording that emitted tones at appropriate intervals. For the first few minutes of the test, the speed was set to 8 km/h and then increased by 0.5 km/h every minute. Children ran in groups of no more than five and were encouraged by a member of the research team. As soon as a child was not able to complete a whole stage, the test was stopped and the child’s score corresponding to the last fully completed stage was recorded. The Maximal Aerobic Speed (MAS) was then calculated. The maximum oxygen intake (VO_2max_) was estimated using the following validation equation: Y = 31.025 + 3.238X − 3.248A + 0.1536AX, where X refers to the MAS and A to the age [29]. The 20 m shuttle-run test has been validated and found to be reliable for the prediction of VO_2max_ in children and adolescents [30] with a good reproducibility in children and adolescents between ages 8 and 18 [29,31,32,33].

#### 2.4.2. Upper-Body Muscular Strength

Upper-body muscular strength was measured with a handgrip dynamometer (Takei Scientific Instruments Co., Ltd., Niigata, Japan). A previous study reported acceptable inter-trial reliability for the hand dynamometer [34]. The participants were asked to adjust the handgrip bar so that the second joint of the fingers was bent to grip the handle of the dynamometer. The participants stood upright with their arms in a vertical position and the dynamometer close to the body. They were then asked to squeeze the hand dynamometer as hard as possible. The test was completed twice, in the left and right arms, and the best recordings of both the arms were averaged. Participants also performed a Medicine Ball Throw test that measures the explosive power of the upper limbs when throwing a medicine ball as far as possible. Participants were seated in a chair with their feet shoulder-width apart and their backs supported so that only upper-body strength was used. The medicine ball was held at shoulder height and thrown forward with a counter movement of the forearms. The test was performed twice, and the research staff recorded the distance between front chair leg and the medicine ball landing site (to the nearest 0.5 cm). The Medicine Ball Throw test has been validated and is reliable for children and adolescents with an ICC = 0.98 [35].

#### 2.4.3. Lower-Body Muscular Strength

Lower-body muscular strength was measured using the Standing Long Jump test. The test was performed on a non-slip hard surface. Participants were asked to jump the longest distance possible from a standing start while swinging both arms. The distance from the take-off line to the point where the back of the heel touched the surface was measured using a measuring tape. The test was conducted twice, and the best score was used [36]. The Counter Movement Jump test was also performed using an Optojump (Microgate Co., New York, NY, USA) [37]. The starting position was a standing position with a straight torso and knees fully extended with the feet shoulder-width apart [37]. Participants were asked to keep their hands on their hips throughout the whole jump. They were instructed to perform a quick downward movement (approximately 90° of knee flexion), and afterward a fast upward movement to jump as high as possible.

### 2.5. Implicit Activity Preferences

The Activity Preference Assessment (APA) is a computerized behavioral task designed to assess and quantify biases in decision making across multiple leisure time activities [13]. The APA is administered on a desktop computer via E-Prime (Psychology Software Tools, Sharpsburg, PA, USA) and takes approximately 10 min to complete. Each participant was first asked how much they like taking part and want to take part in a variety of common physical activities (e.g., ball sports, swimming) and sedentary activities (e.g., arts and crafts, watching TV) using visual analog scales (VAS) to quantify explicit liking and explicit wanting (range 0 to 100). They then complete a forced-choice paradigm, or “would you rather?” game. Out of each pair of activity images (4 sets of 30 pairs, with breaks between sets), they were asked to select, as quickly as possible, the activity they most want to do in order to assess implicit wanting. Sixty-four of the 120 pairs were sedentary vs. physical activities, with the remaining pairs falling within category. Every pair is unique and all possible comparisons were made. Implicit wanting scores from the head-to-head comparisons of sedentary vs. physical activities take into account the choices made and reaction times. These scores were used to compute the bias score (range of −100 to +100), thereby quantifying the relative implicit preference. Positive (+) scores represent a relative preference towards sedentary activities and negative (−) scores represent a relative preference towards physical activities. The data were processed via an automated scoring procedure in Anaconda3 (Austin, TX, USA). The APA was originally developed and validated in English [13], but herein we present data from the French version. All text within the APA and the corresponding participant instructions were translated to French by a bilingual French and English speaker and then back-translated to English to confirm that the original meaning of all the text was retained.

### 2.6. Statistical Analysis

Continuous data are expressed as mean and standard deviation. The assumption of normality was assessed by using the Shapiro-Wilk test. The comparisons between baseline (T0) and after the lockdown and end of restrictions evaluations (T1) were performed using Student’ *t* test or Mann-Whitney test if the assumptions to apply t-tests were not met. The homoscedasticity was analyzed using the Fisher-Snedecor test. Particular attention was paid to the magnitude of T0–T1 differences. The results were expressed using Hedge’s effect size (ES) and 95% confidence interval, and were interpreted according to the rules of thumb reported by Cohen [38], who defined the ES bounds as: small (ES = 0.2), medium (ES = 0.5), and large (ES = 0.8). To analyze the relationships between continuous parameters, Pearson and Spearman correlation coefficients were estimated according to the statistical distribution of variables, and were interpreted as follows: <0.2 negligible, 0.2 to 0.4 weak, 0.4 to 0.7 moderate, >0.7 strong [39]. All statistical analyses were carried out using Stata software (version 15, StataCorp, College Station, TX, USA) for a two-sided Type I error at 5%, with a Sidak’s type I error correction to take into account multiple comparisons.

## 3. Results

A total of 206 healthy-weight kids of ages 9 to 10 participated in the study. At T0, 106 children (9.9 ± 1.03 y) completed the evaluations, including 55 girls (9.9 ± 1.03 y) and 51 boys (9.8 ± 1.06 y). At T1, 100 kids completed the evaluation (9.4 ± 0.6 y), including 47 girls (9.4 ± 0.6 y) and 53 boys (9.4 ± 0.6 y).

**Figure 1:**
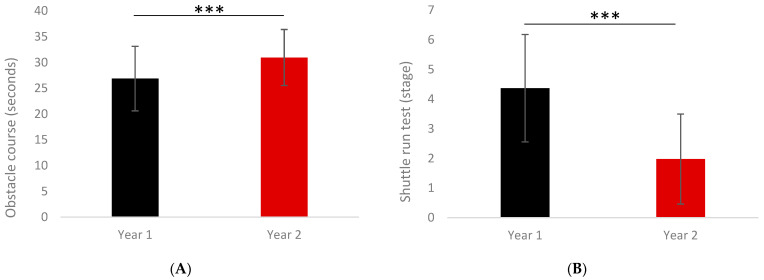
Performances at the Motor Skills Obstacle Course (**A**) and shuttle-run test (**B**) between T0 (year 1) and T1 (year 2). *** *p* < 0.0001.

**Figure 2:**
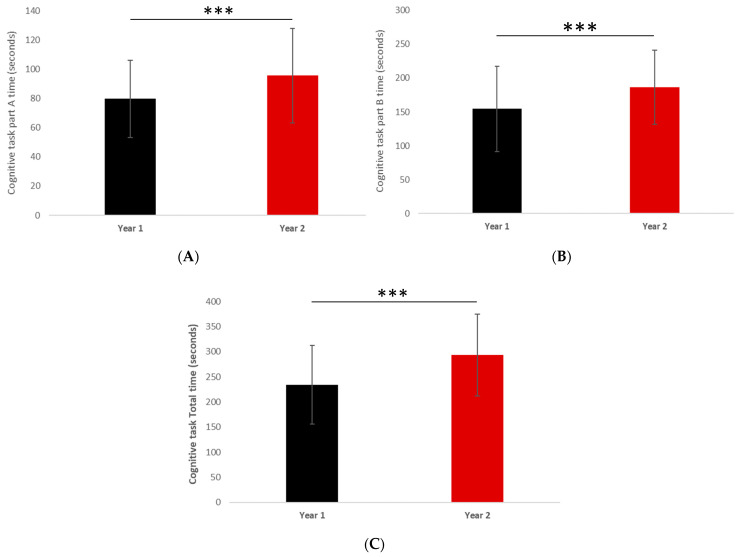
Results of the parts A and B (**A**,**B**) of the Trail Making Test and Total score (**C**) at T0 (year 1) and T1 (year 2). *** *p* < 0.0001.

The participant characteristics, fitness, and executive function scores from T0 and T1 are detailed in Table 1. Weight (kg), BMI (kg/m^2^) and the percentage of fat mass (FM%) did not differ. While there was not significant difference in the Counter Movement Jump height, the performance of the Standing Long Jump was better at T0 (169.9 ± 142.5 cm) compared to T1 (135.2 ± 31.4 cm) (*p* = 0.0367). Regarding physical fitness in the upper limbs, handgrip strength did not differ over time, but Medicine Ball Throw performance declined from T0 to T1 (297.3 ± 81.1 cm vs. 249 ± 52 cm; *p* < 0.0001). The performance of the motor skills test was found to be significantly better (lower execution time) at T0 compared with T1 (26.9 ± 6.2 s vs. 30.9 ± 5.4 s; *p* < 0.0001) (Figure 1A). The shuttle-run test (stages completed), MAS, and estimated VO_2max_ were all significantly lower at T1 compared to T0 (*p* < 0.0001) (Figure 1B). Results from the cognitive functioning task are presented in Table 1 and Figure 2, with better performances seen in part A, part B, and the total score at T0 compared to T1 (*p* = 0.0009; *p* = 0.0008 and *p* < 0.0001, respectively).

There were associations between the TMT total score and the shuttle-run test performance (r^2^ = −0.292; *p* = 0.0043), handgrip strength (r^2^ = −0.279; *p* = 0.0069) and Standing Long Jump performance (r^2^ = −0.221; *p* = 0.0336) at T0. The TMT total score was associated with handgrip strength (r^2^ = −0.366; *p* = 0.0003) and Counter Movement Jump performance (r^2^ = −0.279; *p* = 0.0098) at T1.

A subsample of participants (*n* = 51 at T0; *n* = 51 at T1) completed the APA. Children did not show a difference in explicit liking or wanting for sedentary or physical activities between T0 and T1 (all *p* > 0.05). Similarly, there was not a statistically significant difference in bias scores (relative preference) from the forced-choice task between T0 (−9.5 ± 44.4, physical activity preference) and T1 (6.2 ± 53.5, sedentary preference) (*p* = 0.12).

## 4. Discussion

While there has been a consistently growing body of scientific literature describing an alarming overall decline in physical activity levels and an increase in sedentary time during the COVID-19-related confinement [40], few studies have examined downstream effects on physical fitness and cognitive functioning, especially in children. Prior to the COVID-19 crisis, Tomkimson and collaborators described a reduction in physical fitness by about 25% over the last 40 years in children, which was a function of reduced physical activity levels and increased sedentariness [41]. In line with this trend, our concern is that the unprecedented acceleration of children’s inactivity and sedentariness induced by the lockdown has negatively impacted physical fitness beyond the usual declines over time. Moreover, this public health crisis has resulted in poorer school engagement, which can have detrimental effects on academic performance and executive function. Previous studies have clearly shown that physical fitness, particularly cardiorespiratory fitness, is strongly associated with executive functioning and academic achievement in children and adolescents [21]. Therefore, concomitant declines in these markers can be expected under such extreme circumstances.

In the current study, both muscle strength and cardiorespiratory fitness were significantly reduced among 3rd- and 4th-grade children after the COVID-19 lockdown period compared with pre-pandemic performances. These results are in accordance with previously published findings showing a decline in both cardiovascular [10,11,13] and muscle performances [10] of children and adolescents. Importantly, while the previous studies assessed physical fitness in the same participants before and after, the time intervals between their two evaluations were larger than the lockdown periods, and therefore did not isolate the effects of the consecutive COVID-19-related restrictions.

The present study is the first to also question the potential effect of the pandemic mitigation measures on children’s activity preferences. Using a computerized behavioral task designed to assess and quantify biases in decision making across multiple physical and sedentary activities [13], our results suggest that despite a clear decline in physical activity levels [40], increase in sedentary time [40], and reduction in overall fitness, the explicit liking and wanting were not detrimentally affected. Though there was a slight shift from a mean preference for physical activities towards a mean preference for sedentary activities, the difference in bias scores was not statistically significant. Indeed, scores at T0 and T1 were close to 0, indicating an equal preference. This result is encouraging, indicating that the observed deleterious effects of the lockdown on movement behaviors and physical health might not be irreversible, and that children might be willing to re-engage in active behaviors, pending adapted and appropriate interventions and messaging.

Although our results show only modest correlations between fitness and executive function task outcomes both at T0 and T1, we observed an alarmingly lower cognitive performance in 3rd- and 4th-grade children after the confinement compared with age-matched participants before the public health restrictions. As previously described, the stress induced by the sanitary crisis [18,19,20] as well as the lower intellectual stimulation induced by a reduction in school time and the use of virtual classes could explain such a decline. Importantly, while some studies showed that this confinement-related cognitive decline was more pronounced among children from disadvantaged socio-economic backgrounds [17], 50% of the re-enrolled classes of the present study were located in low socio-economic areas and 50% in high socio-economic areas, and no differences were observed between these school-types. In other words, our results suggest that the negative impact of the lockdown on children’s executive function might have been independent of the socio-economic origin of the kids. It is also possible that the alarming reductions in children’s healthy movement behaviors, combined with the decline in their physical fitness, might have directly impacted their executive and cognitive abilities, but additional studies are needed [21,22]. The modest correlations between fitness and performance of the cognitive task in the current study may be explained by the indirect nature of the physical test and the sample size.

The present work contributes to the international literature regarding the detrimental effects of the COVID-19 pandemic on multiple inter-related physical and cognitive health outcomes, with new evidence on French schoolchildren. However, our results must be interpreted in light of some limitations. First, despite the fact that the children at T0 and T1 were matched for age, gender, and their socio-economic statuses, and that the evaluations were performed in the same schools, the samples remain modest for school-based work and larger studies would help to clarify the interactions amongst health behaviors and outcomes. Additionally, although all the field tests that were used have been validated in children and adolescents, direct laboratory-based evaluation of fitness would have provided more objective results. Finally, the children in the sample had predominantly healthy weights, and therefore we cannot generalize these findings to children with obesity. However, studies in adults suggest that the negative effects of the lockdown on health behaviors disproportionately affected those with obesity [42].

## 5. Conclusions

To conclude, the present work highlights an alarming decline in both overall physical fitness and cognitive performance among primary school French children due to the public health restrictions imposed in order to slow down the spread of the COVID-19 virus. In concordance with the existing evidence describing the deleterious effects of the confinement on our children’s movement behaviors and overall health, our results reinforce the need to urgently develop preventive strategies in anticipation of further mitigation measures. We must work to encourage a healthy, active lifestyle in children in order to preserve their physical, mental, and social health during this continued public health crisis.

## Figures and Tables

**Table 1:** Anthropometric characteristics, physical fitness, and cognitive functioning results between T0 and T1.

	T0		T1			
	Mean	SD	Mean	SD	*p*	ES
Weight (kg)	32.96	8.71	35.13	10.26	0.1862	−0.22 [−0.56; 0.11]
BMI (kg/m^2^)	17.23	3.51	18.20	3.94	0.1425	−0.25 [−0.59; 0.09]
FM (%)	24.23	7.20	24.41	6.85	0.9031	−0.02 [−0.42; 0.37]
CMJ (cm)	18.01	4.60	17.36	3.51	0.3213	0.15 [−0.15; 0.46]
SBJ (cm)	169.87	142.57	135.23	31.40	0.0367	0.33 [0.01; 0.64]
Handgrip (N)	14.30	3.54	14.02	3.04	0.5892	0.08 [−0.22; 0.39]
TMB (cm)	297.28	81.11	249.06	52.13	<0.0001	0.70 [0.38; 1.01]
Motor skills (s)	26.88	6.26	30.97	5.43	<0.0001	−0.69 [−1.01; −0.37]
MAS (km/h)	9.68	0.90	9.00	0.75	<0.0001	0.80 [0.48; 1.12]
VO_2max_ (mL/min/kg)	45.66	4.25	43.05	3.69	<0.0001	0.65 [0.33; 0.96]
Shuttle run stage	4.37	1.81	1.98	1.52	<0.0001	1.41 [1.06; 1.75]
TMT-A (s)	79.69	26.47	95.60	32.36	0.0009	−0.53 [−0.85; −0.22]
TMT-B (s)	154.43	62.95	186.44	54.63	0.0008	−0.53 [−0.85; −0.22]
TMT-Tot (s)	234.12	77.90	293.24	80.98	<0.0001	−0.74 [−1.05; −0.41]

T0: Baseline; T1: Time 1; BMI: Body Mass Index; CMJ: Counter Movement Jump; ES: Effect Size; FM: Fat Mass; MAS: Maximal Aerobic Speed; *p*: *p* value; SBJ: Standing Broad Jump; SD: Standard Deviations; TMB: Throw Medicine Ball; TMT: Trail Making Test.

## Data Availability

Not available.
